# Using Iron L-Edge and Nitrogen K-Edge X-ray Absorption Spectroscopy to Improve the Understanding of the Electronic Structure of Iron Carbene Complexes

**DOI:** 10.1021/acs.inorgchem.4c01026

**Published:** 2024-06-27

**Authors:** Meiyuan Guo, Robert Temperton, Giulio D’Acunto, Niclas Johansson, Rosemary Jones, Karsten Handrup, Sven Ringelband, Om Prakash, Hao Fan, Lisa H. M. de Groot, Valtýr Freyr Hlynsson, Simon Kaufhold, Olga Gordivska, Nicolás Velásquez González, Kenneth Wärnmark, Joachim Schnadt, Petter Persson, Jens Uhlig

**Affiliations:** † Division of Chemical Physics, Department of Chemistry, Lund University, 22100 Lund, Sweden; ‡ MAX IV Laboratory, Lund University, 22100 Lund, Sweden; § Division of Synchrotron Radiation Research, Department of Physics, Lund University, 22100 Lund, Sweden; ∥ NanoLund, Lund University, 22100 Lund, Sweden; ⊥ Centre for Analysis and Synthesis (CAS), Department of Chemistry, Lund University, 22100 Lund, Sweden; # Division of Computational Chemistry, Department of Chemistry, Lund University, 22100 Lund, Sweden; ∇ LINXS Institute of Advanced Neutron and X-Ray Science, Lund University, 22370 Lund, Sweden; ¶ Department of Chemical Engineering, Stanford University, 94305 Stanford, California, United States

## Abstract

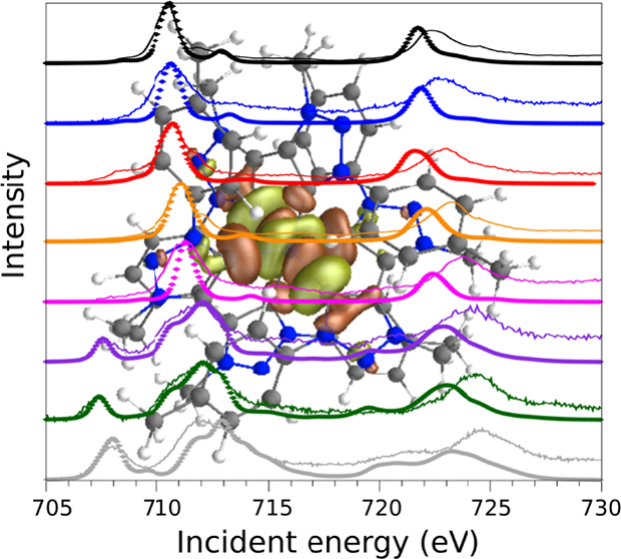

Iron-centered N-heterocyclic carbene compounds have attracted much attention in recent years due to their long-lived excited states with charge transfer (CT) character. Understanding the orbital interactions between the metal and ligand orbitals is of great importance for the rational tuning of the transition metal compound properties, e.g., for future photovoltaic and photocatalytic applications. Here, we investigate a series of iron-centered N-heterocyclic carbene complexes with +2, + 3, and +4 oxidation states of the central iron ion using iron L-edge and nitrogen K-edge X-ray absorption spectroscopy (XAS). The experimental Fe L-edge XAS data were simulated and interpreted through restricted-active space (RAS) and multiplet calculations. The experimental N K-edge XAS is simulated and compared with time-dependent density functional theory (TDDFT) calculations. Through the combination of the complementary Fe L-edge and N K-edge XAS, direct probing of the complex interplay of the metal and ligand character orbitals was possible. The σ-donating and π-accepting capabilities of different ligands are compared, evaluated, and discussed. The results show how X-ray spectroscopy, together with advanced modeling, can be a powerful tool for understanding the complex interplay of metal and ligand.

## Abstract 2

The measurement and advanced modeling of soft X-ray absorption spectra from both the ligand and the metal center allowed a unique view of the orbitals inside complex molecules and the extraction of descriptive parameters.

## Introduction

The development of sustainable, environmentally friendly, efficient, long-living, and scalable systems for solar energy harvesting is one of the biggest challenges of our time.^1^ The generation, extraction, and usage of charge carriers, created with the energy of a single absorbed photon, are at the core of a molecular solar energy harvester.^2^ In recent years, molecular light-harvesting complexes have seen a strong recurrence through several new approaches for the tuning of molecular states.^3−11^ Promising photophysical properties such as luminescence and electron transfer are obtained by tuning the energies of specific states that are involved in unfavorable de-excitation pathways by selectively using the σ-donating and π-accepting capabilities of specific ligands.^3,12−15^ This manipulation of the energy levels eliminates fast recombination processes and extends the lifetime of the charge-separated states from several picoseconds in the first complexes in the series to nanoseconds in the latest.^3,4^ The ligand manipulation has also resulted in some of the first stable, nearly perfect octahedral Fe^III^ and Fe^IV^ complexes with interesting photophysical properties.^15,16^

**Figure 1:**
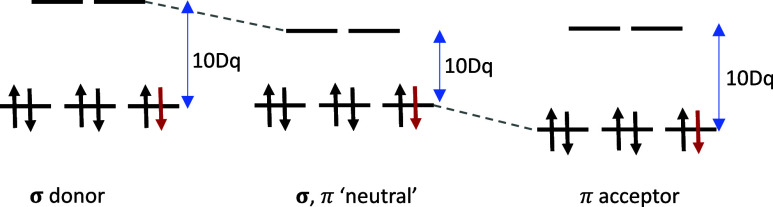
Scheme that illustrates the effects of σ donating and π-back-bonding on the frontier orbital energies.

The introduction of strong σ-donating N-heterocyclic carbene (NHC) ligands in [Fe^II^(btz)_2_(bpy)]^2+^ (bpy = 2,2′-bipyridine, btz = 3,3′-dimethyl-1,1′-bis(*p*-tolyl)-4,4′-bis(1,2,3-triazol-5-ylidene)) results in a long-lived triplet metal-to-ligand charge-transfer (^3^MLCT) state of 13 ps compared to the 130 fs lifetime of the ^3^MLCT state of [Fe(bpy)_3_]^2+^.^12^ Recent time-resolved X-ray emission spectroscopy (XES) investigations have confirmed a deactivation pathway from a hot ^3^MLCT state to the triplet metal-centered (^3^MC) state, in competition with vibrational cooling of the ^3^MLCT state.^17^ An extra ligand replacement from bpy to btz significantly increases the ^3^MLCT lifetime to 528 ps.^14^ These achievements demonstrate the remarkable capability of the NHC ligands to block the rapid deactivation of the ^3^MLCT state via meter-centered (MC) states. Other groups found a similar correlation between the NHC ligand count and the position of the ^3^MC and ^3^MLCT states.^11^ Hence, it is important to probe the metal-character molecular orbitals, which are directly correlated to the MC states. A theoretical investigation on how the different character ligands affect the relative energies between MC states and ^3^MLCT state was carried out, which provided a basis for the design of novel carbene complexes with promising photophysical properties.^18,19^ The general effect of σ donation and π-back-bonding on the frontier orbital energies in octahedral complexes is illustrated in Figure 1. With increasing σ donation, the typically metal-centered e_g_ levels of the iron orbitals are pushed up, which is visible in a shift of the e_g_ resonance to higher binding energies and an increase of the 10Dq value (the measure of the ligand field-induced splitting between the t_2g_ and e_g_ orbitals). An increasing π-back-bonding also increases the 10Dq values while maintaining a constant e_g_ level.

Directly studying the ligand field states using optical spectroscopy can be challenging due to the many potential transitions involved. The parity-forbidden d–d transitions between metal-centered orbitals are generally weaker than the typically intense ligand-to-metal charge transfer (LMCT) or metal-to-ligand charge transfer (MLCT) features. Element-specific X-ray spectroscopy uses a defined core level as the initial or final state of the transitions, which simplifies the identification of specific states. For the study of iron carbene-based complexes, X-ray absorption spectroscopy with transitions from the 2p and 1s (L- and K-edge XAS, respectively),^11,20−23^ Fe Kα and Kβ X-ray emission spectroscopy (XES),^11,21,22^ and resonant techniques such as resonant inelastic X-ray scattering (RIXS) have been used to track the transitions and energy levels.^24^ Each of these X-ray spectra is highly sensitive to specific aspects of the electronic and geometric structures, and a (partial) combination of them allows a complete description of the metal character orbitals.^25^ The high-energy resolution fluorescence detected X-ray absorption near edge structure (HERFD-XANES) at iron K pre-edge has been used to understand the orbital interactions between metal and ligands for iron NHC complexes.^11,20^ A correlation between the number of NHC ligands and the electronic structure of iron carbene compounds was established through the investigation of a combination of HERFD-XANES and XES.^11^ We have used time-resolved XES investigations of [Fe^II^(btz)_2_(bpy)]^2+^ to establish the deactivation pathways from a hot ^3^MLCT state to the triplet metal centered (^3^MC) state, in competition with vibrational cooling of the ^3^MLCT state.^17^ The time-resolved XES combines an X-ray solution scattering (XSS) study on [Fe(bmip)_2_]^2+^ (bmip = 2,6-bis(3-methyl-imidazole-1-ylidine)-pyridine), which identified a metal-centered triplet (^3^MC) by probing the spatial extension of the electronic orbitals and the evolving molecular structure.^22^ In combination with advanced calculation tools, these techniques improve our understanding and can provide a basis for the conscious design of novel iron compounds with targeted photophysical properties.

**Figure 2:**
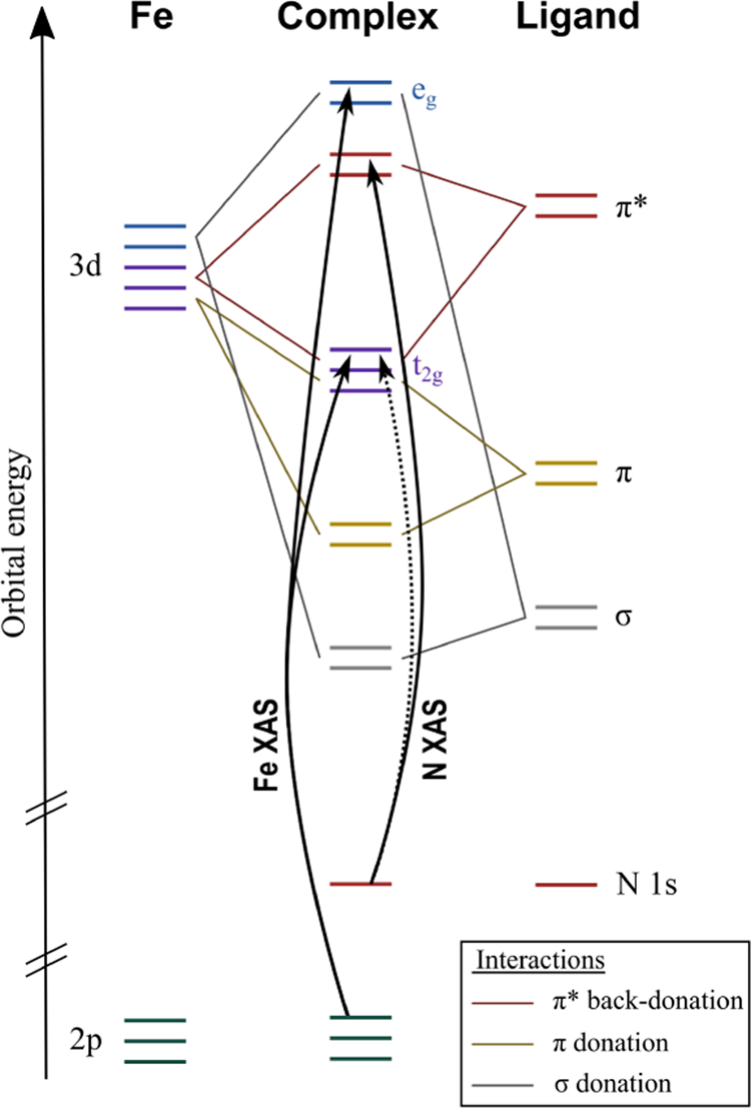
Scheme of the metal–ligand interactions in the quasi-octahedral ligand field-splitting convention. The specific core-to-valence transitions probed in the Fe L-edge and N K-edge XAS experiments are highlighted.

**Figure 3:**
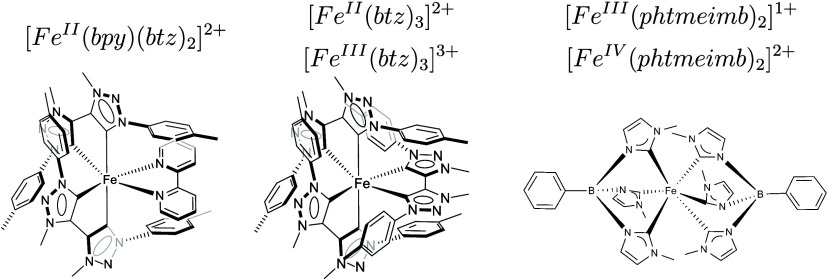
Molecules discussed in this paper.

In this article, we use the combination of Fe L-edge XAS and N K-edge XAS to investigate the metal–ligand orbital interaction for a series of iron N-heterocyclic carbene complexes with different oxidation states. The complexes [Fe^II^(btz)_2_(bpy)]^2+^, [Fe^II^(btz)_3_]^2+^, and [Fe^III^(btz)_3_]^3+^ with bidentate ligands and the two complexes [Fe^III^(phtmeimb)_2_]^1+^, and [Fe^IV^(phtmeimb)_2_]^2+^ with tridentate ligands have different oxidation states of the iron atoms and are illustrated in Figure 3.^12−16^ Metal L-edge XAS probes transitions into valence orbitals that overlap with metal core orbitals, i.e., the t_2g_ orbital and the e_g_ orbitals. The ligand K-edge XAS, through nitrogen 1s excitations, provides a complementary probe of orbitals that are primarily located on the ligand but may be hybridized with metal states. For example, transitions from the N 1s to the metal-centered t_2g_ orbitals are accessible if the delocalized π system of the ligand couples to the iron orbitals via back-donation, as indicated by the dashed arrow in Figure 2. X-ray absorption can, in many cases, directly probe the strength of the π back-donation through electron excitations into the empty ligand character orbitals that overlap with the metal core orbitals. One then usually speaks about the orbitals having some metal 3d character (Figure 2).

Description of the σ and π donation can be carried out through the edge position and the integrated intensity of the corresponding transitions in the XA spectrum.^26^ The quantitative evaluation of the ligand field splitting is relatively straightforward for systems that have both the t_2g_ and e_g_ holes. In a low-spin d^6^ system where t_2g_ is filled, additional information must be obtained either from other techniques such as X-ray-induced photoelectron emission (XPS) or a direct two-photon process such as X-ray emission spectroscopy (XES) must be used that can measure both the occupied and unoccupied orbitals. An advanced example of the latter is 2p 3d resonant inelastic X-ray scattering (RIXS), which has been used to answer this question for similar complexes but has, as a photon-hungry technique, much higher demands on X-ray flux and sample stability.^23,27−29^

We compare experimental XA spectra to simulated spectra obtained by restricted active space (RAS), multiplet calculations, and time-dependent density functional theory (TDDFT). The spectral components, relative intensities, and energy positions were interpreted using orbital composition analysis. The ligand character of electron-donating, -accepting, and ligand field splitting have been evaluated through the charge-transfer multiplet calculations. The combination of Fe L-edge and N K-edge XAS provides complementary information about the orbital interactions between the metal center and ligands. These investigations give direct insight into the effects of these different ligands. Of particular interest might be the direct visualization of the effects of the σ-donating and π-back-bonding ligand nature on the t_2g_ and e_g_ metal-centered levels. In a previous publication, we discussed such effects for another series of tridentate ligands but only for low-spin ferrous (Fe^II^) iron oxidation states.^23^ In the Supporting Information, we extend the discussion in the present paper to the tridentate ligand discussed in the previous project for comparison (see Figure SI 1).

## Experimental Details

The experimental X-ray absorption (XA) spectra were collected during several experiments, with most of the spectra measured at the HE-SGM beamline of the BESSY II synchrotron radiation facility at Helmholtz-Zentrum Berlin. This beamline, operating in the 200 to 800 eV energy range, produces a 1 mm × 200 μm X-ray spot on the sample that was scanned during the experiment to avoid sample damage. A partial electron yield detector with a retardation potential of 150 and 470 V for N K-edge and Fe L-edge XAS measurements, respectively, was used to collect the photoelectrons. The supporting photoelectron spectroscopy was measured using a Scienta R3000 hemispherical analyzer. The samples were prepared in a nitrogen atmosphere glovebox by spin- or drop-casting a deoxygenated and dry solution with acetonitrile onto a gold surface. After the solvent evaporated, the samples were transferred into an ultrahigh vacuum for measurement within 5 min.

The photon energy used in the N K-edge XAS experiments was calibrated using the difference in kinetic energy between photoemission peaks generated by first- and second-order light transmitted through the monochromator of the beamline. The energies of the Fe L-edge spectra were calibrated to the nearby F K-edge of the PF_6_^–^ counterions, where the first and dominant XAS feature was set to have a photon energy of 691.5 eV. The shape of the F K-edge XAS background generated by the PF_6_^–^ counterions was extracted from the difference XA spectrum measured over the F K-edge and Fe L-edge region of [Fe(bpy)_3_]^2+^ with PF_6_^–^ and Cl^–^ counterions and then subtracted from the data.

## Computational Details

The geometries used for the XAS calculations are based on DFT optimizations, which were published in our previous work.^12,13,15,16,30^

### RAS Calculations of Fe L-Edge XAS

The calculations of iron L edge XA spectra were performed using the restricted-active space (RAS) method with atomic natural orbital-relativistic core-correlated basis set ANO-RCC-VDZ through OpenMolcas.^31^ The method for metal L-edge XAS has previously shown its validity for 3d transition metal complexes.^32−41^ The active space used for spectral calculations is designated as RAS(*n*, *l*; *i*, *j*), where *i* and *j* are the numbers of orbitals in the subspaces RAS1 and RAS2, respectively, *n* is the total number of electrons in the active space, and *l* is the maximum number of holes allowed in RAS1. For the metal L-edge XAS calculation, the Fe 2p core orbital is placed in RAS1, while the five orbitals with metal 3d character together with two ligand-character σ-donation orbitals and three empty orbitals of π symmetry are placed in RAS2. For Fe^II^ complexes, the active space is RAS(16,1;3,10); for Fe^III^ complexes, it is RAS(15,1;3,10); and for the Fe^IV^ complex, it is RAS(14,1;3,10). This active space has been used successfully to describe the features in iron K-edge XAS, Fe Kα XES, and Fe 2p3d RIXS of iron carbene complexes.^20,22,23^ The orbital composition analyses on selected active orbitals were performed through a modified Mulliken population analysis^42^ using the Multiwfn program^43^ based on the orbital wave function from RAS calculation.

The restricted active space self-consistent field (RASSCF) wave function optimizations were performed using the state average (SA) formalism,^44^ which means that the same orbitals are used for all states of a specific spin and symmetry. Scalar relativistic effects were included by using a second-order Douglas–Kroll–Hess Hamiltonian,^45,46^ in combination with the ANO-RCC basis set and the use of a Cholesky decomposition approach to approximate the two-electron integrals.^47−49^ The dependence of the Fe L-edge XA spectral features on the number of core-excited states has been checked by increasing the number of final states gradually; the spectra calculated with increasing number of final states are available in the Supporting Information.

The spin–orbit coupling is included in the RAS state-interaction (RASSI) approach.^50,51^ For comparison to the experimental spectra, the simulated RAS spectra were convoluted with a Gaussian broadening of 0.3 eV and a Lorentzian broadening with a full width at half-maximum (fwhm) of 0.4 and 0.8 eV for the Fe L_3_ and L_2_ edge, respectively. The same shift energy of 4.63 eV is applied for all calculated iron L-edge XA spectra. Spectral plots with individual energy shifts are available in the Supporting Information.

### Multiplet Calculations of Fe L-Edge XAS

The multiplet calculations of iron L-edge XAS were carried out first under a crystal field multiplet (CFM) level (without including the metal–ligand orbital covalent mixing), and then the spectra were improved at the charge-transfer multiplet (CTM) level by including additional configurations of ligand–metal charge transfer (LMCT) and metal–ligand charge transfer (MLCT) to describe covalent metal–ligand interactions. All of the multiplet calculations were carried out using the QUANTY software.^52,53^ The additional configurations of LMCT and MLCT allow the description of σ, π donation and π back-donation, respectively. The CFM parameters (10Dq, Dt, and Ds) are extracted from the *Ab initio* ligand-field theory (AILFT) calculations using ORCA.^54,55^ The AILFT calculations are performed by using the complete active space self-consistent field (CASSCF) method with *n* electrons (*n* = 4, 5, 6) and five orbitals (*n*,5) in the active space. The CASSCF was then refined by an N-electron valence second-order perturbation treatment of the dynamic correlation. The full sets of states with a total of 50 singlets, 45 triplets, and 5 quintets were calculated for the 3d^4^ (Fe^IV^) and 3d^6^(Fe^II^) complexes. The 3d^5^ complex was described as having an active space (5, 5), and a total of 75 doublets, 24 quartets, and 1 sextet were included in the calculation.

In addition to the parameters used for the CFM calculations, the CTM calculations require additional parameters that describe the metal–ligand interactions. These additional parameters are as follows: charge-transfer energies of the (Δ_LMCT_ and Δ_MLCT_), the energy difference (10Dq_L_) between σ and π donating orbitals, and three charge-transfer integrals (V_Lσ(e_g_)_, V_Lπ(t_2g_)_, and V_Lπ*(t_2g_)_) for each metal–ligand orbital interactions. These CTM parameters are evaluated by approximating the Fe 3d partial density of states (PDOS) from DFT calculations with the LDA functional. The Fe 3d PDOS of the ground state and the core-ionized state in the Z + 1 approximation (for parameters in the core-excited configuration) are available in the SI. The CTM parameters were calculated following the procedure used for similar complexes described in the recent paper by Kunnus et al.^23^ We summarize the parameters used for multiplet calculations in Table 1.

**Table 1:** Parameters for Multiplet Calculations^a^

	[Fe^II^(btz)_2_(bpy)]^2+^	[Fe^II^(btz)_3_]^2+^	[Fe^III^(btz)_3_]^3+^	[Fe^III^(phtmeimb)_2_]^1+^	[Fe^IV^(phtmeimb)_2_]^2+^
10Dq	2.7/2.5	3.2/2.8	3.3/2.9	3.8/3.5	3.6/3.1^b^
Ds	0.026/0.26	–0.008/–0.008	0.005/0.005	0.004/0.004	–0.006/0.004^b^
Dt	0.044/0.024	0.031/0.025	0.018/0.017	0.015/0.013	–0.14/–0.15^b^
Δ_LMCT_	3.8/2.8	3.4/2.4	3.4/2.4	3.2/2.2	3.2/2.2^b^
Δ_MLCT_	3.4/4.4	2.2/3.2	2.4/3.4	2.8/3.8	3.0/4.0^b^
10Dq_L_	1.3/1.6	1.2/1.1	1.2/1.1	1.2/1.4	1.0/0.9^b^
V_L_σ_(e_g_)_	2.1/1.8	2.8/2.6	3.0/2.8	3.7/3.3	3.6/3.2^b^
V_Lπ(t_2g_)_	0.8/0.6	0.8/0.6	1.0/0.8	1.4/1.2	2.2/2.1^b^
V_Lπ*(t_2g_)_	0.95/0.8	1.8/1.6	1.5/1.3	1.4/1.2	1.2/1.0^b^

a The first value in a cell corresponds to the ground-state configuration and the second to the core-excited configuration.

b The parameters used for [Fe^IV^(phtmeimb)_2_]^2+^ were estimated from the values calculated for [Fe^III^(phtmeimb)_2_]^1+^. The CASSCF/NEVPT2 in AILFT of [Fe^IV^(phtmeimb)_2_]^2+^ predicts a wrong spin state as the ground state and was thus rejected.

### N K-Edge XAS Calculations

The Nitrogen K-edge XA spectra were calculated using the TD-DFT method as implemented in ORCA using the B3LYP functional and the 6-31g* basis set.^55^ The resulting N K-edge XA spectra were shifted to higher energies by 11.83 eV, which is the average value of the five individual shift energies that would be needed to match the data. The underestimation of the excitation energy comes from the limitation of DFT in describing potentials near the nucleus. Thus, the N 1s core orbitals are wrong in energy. However, the TD-DFT approach that is used here provides accurate relative excitation energies and intensities that allow a direct comparison to the energy shifts observed in the experiment. The relative excitation energies of the peaks are mainly determined by the molecular valence orbitals and are less affected by the uncertainties of the core orbitals. The calculated spectra have been broadened with a Gaussian function with a full width at half-maximum (fwhm) of 0.4 eV.

## Results and Discussion

### Fe L-Edge XAS: Probing the Metal Center

**Figure 4:**
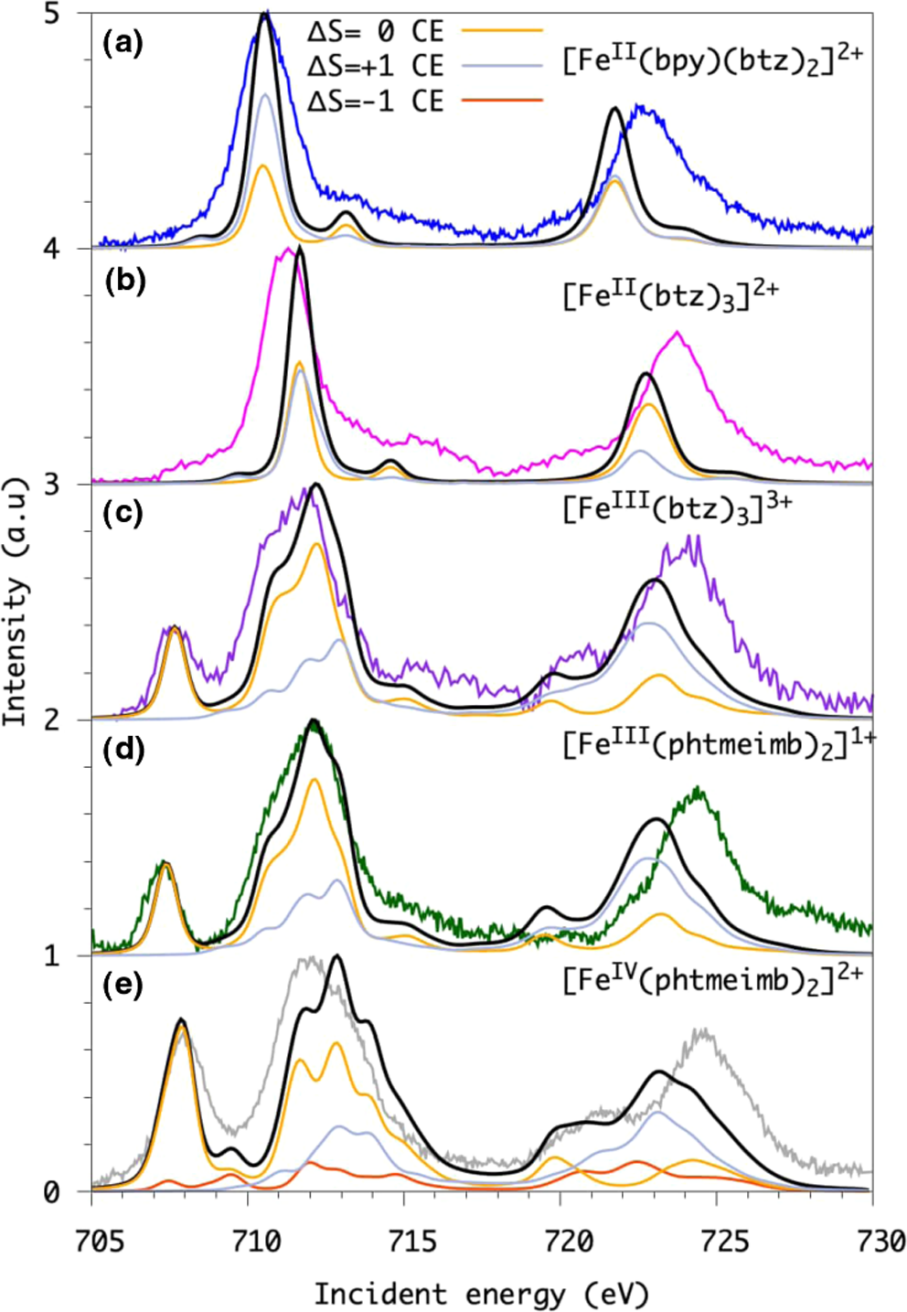
Calculated, measured, and spin-decomposed Fe K-edge XA spectra sorted after the final core-excited (CE) spin state. From Top to bottom: The first two Fe^II^ (d^6^) systems have a low-spin singlet ground state, and we show the singlet(Δ*S* = 0) and triplet (Δ*S* = 1) core-excited state. The next two systems have a Fe^III^ (d^5^) low-spin doublet ground state, and we show the doublet (Δ*S* = 0) and quartet (Δ*S* = 1) core-excited state. The intermediate-spin Fe^IV^ (d^4^) complex at the bottom has a triplet ground state, and we show the singlet (Δ*S* = −1), triplet (Δ*S* = 0), and the quintet (Δ*S* = 1) core-excited state. (From top to bottom (a) [Fe^II^(btz)_2_(bpy)]^2+^, (b)[Fe^II^(btz)_3_]^2+^, (c) [Fe^III^(btz)_3_]^3+^, (d) [Fe^III^(phtmeimb)_2_]^1+^, and (e) [Fe^IV^(phtmeimb)_2_]^2+^).

The experimental Fe L-edge XA spectra are presented in Figure 4. The Fe L-edge XA spectrum is split into the L_3_ and L_2_ edges by Fe 2p spin–orbit splitting. This paper’s spectral feature comparison and discussion will focus on the L_3_ edge, as it is better resolved than the L_2_ edge, mainly due to the difference in lifetime broadening.^56,57^

The two Fe^II^ compounds [Fe^II^(btz)_2_(bpy)]^2+^ and [Fe^II^(btz)_3_]^2+^ have a singlet ground state. The L_3_ edge XA spectra of these compounds are dominated by a single main peak, which originate from the electron excitation from the Fe 2p core level into empty molecular orbitals with an e_g_ character. These peaks are located at 710.6 eV in the case of [Fe^II^(btz)_2_(bpy)]^2+^ and at 711.30 eV in the case of [Fe^II^(btz)_3_]^2+^. There are some features at the higher-energy side of the L_3_ edge due to excitations into the empty ligand-character π*-back-bonding orbitals. They correspond to a back-donation charge transfer. For the singlet ground-state complexes, there are still some observable features on the lower-energy side of the e_g_ main peak, although there is no hole in the t_2g_ orbitals. The occurrence of this intensity mechanism will be explained later based on the RAS calculations. The Fe^III^ and Fe^IV^ compounds, [Fe^III^(btz)_3_]^3+^, [Fe^III^(phtmeimb)_2_]^1+^ and [Fe^IV^(phtmeimb)_2_]^2+^, have an extra, well-separated peak at the lower-energy side of the main L_3_ feature. Its separation from the e_g_ peak and its intensity are very pronounced in comparison to what is visible in the XA spectra of the low-spin Fe^II^ compounds. The reason is/are the extra hole(s) in the t_2g_ orbital. [Fe^III^(phtmeimb)_2_]^1+^ has a slightly lower t_2g_ peak in comparison to that of [Fe^III^(btz)_3_]^3+^, while it has a slightly higher-energy e_g_ peak. The t_2g_ peak of [Fe^IV^(phtmeimb)_2_]^2+^ is more intense than that for Fe^III^ compounds due to the additional t_2g_ hole.

The experimental Fe L-edge XA spectra are calculated with two different approaches using the RAS method and multiplet calculations. Both RAS and multiplet theory reproduce the general experimental spectral features in terms of peak intensity and relative positions; however, they allow for interpretation from different perspectives. We mainly use RAS calculations to understand the origin of the X-ray spectral features through contribution analysis in terms of orbital transitions and spin states. The multiplet calculations (CFM and CTM) are used to evaluate the ligand or metal characters of the orbitals and thus their charge donating and accepting capabilities.

**Figure 5:**
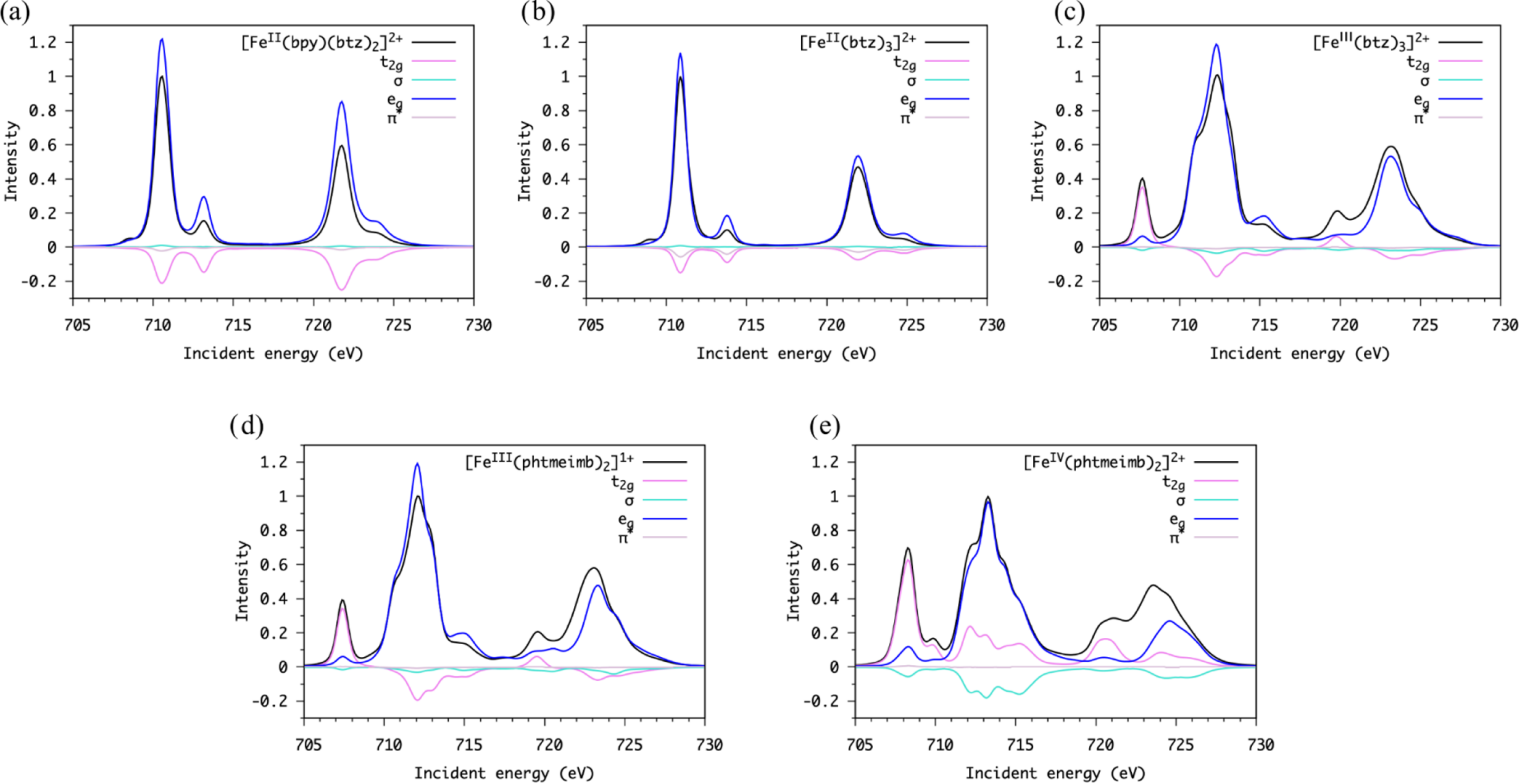
Decomposition of the calculated Fe L-edge spectrum (black solid line) into molecular orbital contributions. The ’negative’ and ’positive’ intensity correspond to values of the orbital occupation loss and gain that are correlated with a change in the transition strength. This change originates from the core electron excitations and can include simultaneous valence electron excitations.^34^ (a) ([Fe^II^(btz)_2_(bpy)]^2+^, (b) [Fe^II^(btz)_3_]^2+^, (c) [Fe^III^(btz)_3_]^3+^, (d) [Fe^III^(phtmeimb)_2_]^1+^, and (e) [Fe^IV^(phtmeimb)_2_]^2+^).

The RAS calculations allow for the analysis of the XA spectra in terms of core-excited states with specific spin multiplicities, as shown in Figure 4. In the final-state contribution analysis, Δ*S* refers to changes in the total spin angular momentum. For a triplet ground state, Δ*S* = +1 and Δ*S* = −1 represent quintet and singlet states, respectively. The calculated spectral features can also be interpreted through the orbital occupation difference between the ground- and core-excited states; see Figure 5. The intensity in the absorption spectra is calculated from the ground state to a final state with a specific spin–orbit coupling. These final states are formed through linear combinations of spin-free states with individual mixing weights. Each spin-free state has known orbital occupation numbers in each active orbital. In the orbital contribution analysis, only the spin-free states contributing to the final intensity of a transition between spin–orbit coupled states were considered. Due to the large number of transitions calculated for the spectrum, it is not desirable to analyze each transition. Instead, a representation that adds all contributions is used. The negative intensities of individual orbital contributions are not the exact intensities, but were derived from the orbital occupation difference between the initial and final state. The electron loss in the Fe 2p core orbital is not shown in this figure, as there is always one electron lost in the core orbital in all cases. The purpose of combining similar transitions into a spectrum is to provide a chemically intuitive molecular-orbital picture. The original theoretical description of the orbital contribution analysis is available in our previous paper.^34^

For [Fe^II^(btz)_2_(bpy)]^2+^ and [Fe^II^(btz)_3_]^2+^, the calculated XA spectra agree well with the experimental spectra; in particular, the features on the higher- and lower-energy sides of the main peak are present. From the orbital contribution analysis (Figure 5a,b), it can be inferred that the main peak intensities are dominated by electron excitation into the empty e_g_ orbitals. Some additional d–d transitions (from the occupied t_2g_ to e_g_ orbitals) along with the core–electron excitations exist. The intensity and energy of the feature on the higher energy side of the main peak edge are underestimated. The correct determination would require the involvement of more back-donation charge-transfer states in the set of final states, and it would significantly increase the computational time and beyond the RAS capability for these large compounds.

The observed absorption intensity at the higher energy side mainly stems from the multiple-state excitations into the valence orbitals along with direct core excitations, which is confirmed by the orbital contribution analysis. As aforementioned, there are some observable features on the lower-energy side of the L_3_ main peak in the experimental spectra of [Fe^II^(btz)_2_(bpy)]^2+^ and [Fe^II^(btz)_3_]^2+^ (around 708.5 to 709 eV), although there is no hole in the t_2g_ orbitals. One possibility is that these features are intensity contributions with significant contributions from triplet core-excited states with parallel 2p and 3d orientation and characterized by significant spin–orbit coupling effects but can gain intensity through spin–orbit coupling in the core-excited states.^38,39^ The spin contribution decomposition analysis shows that the pre-edge features indeed could come from a triplet core-excited final state (Δ*S* = +1); see Figure 4.

**Figure 6:**
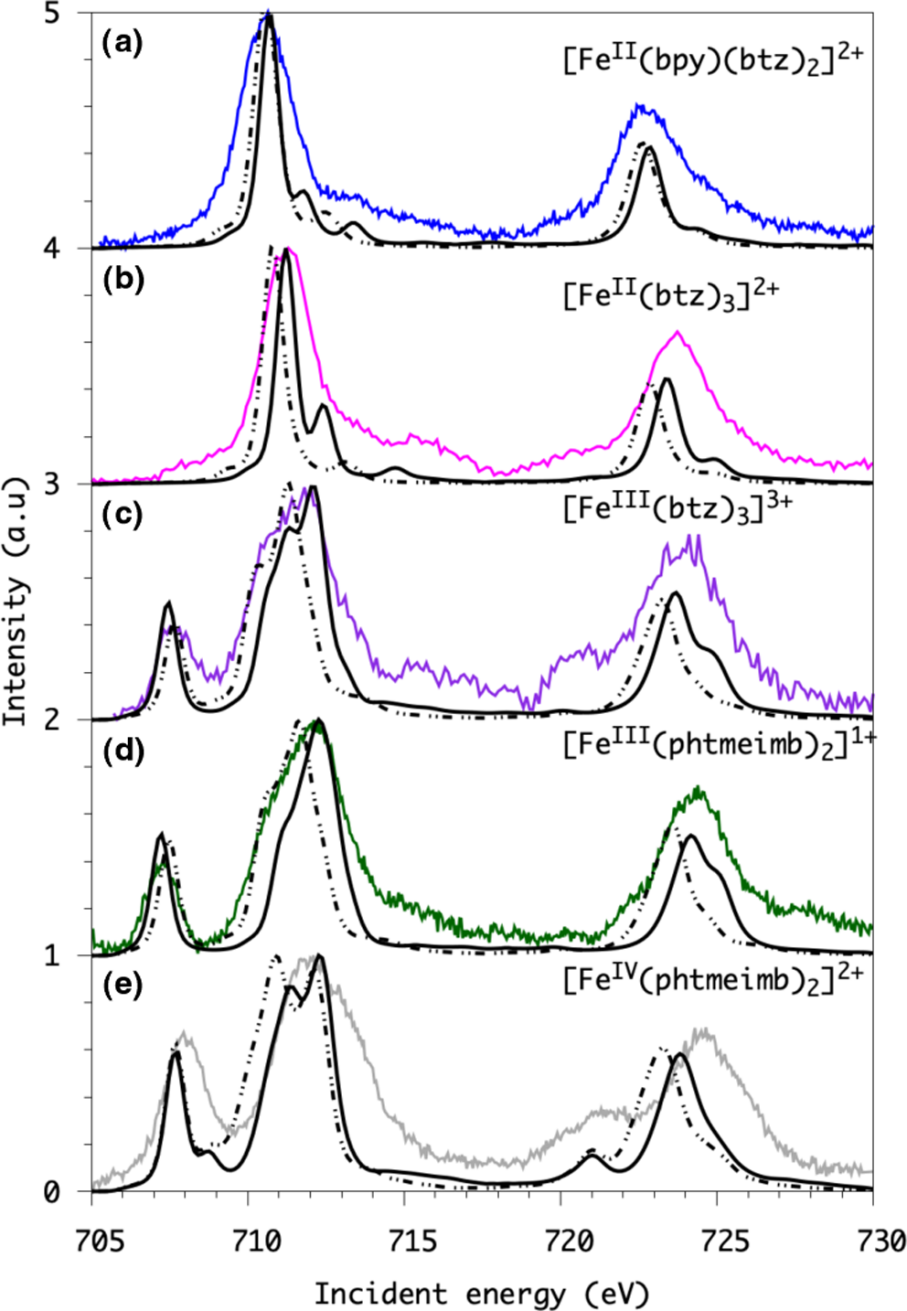
Multiplet calculations of Fe L-edge XAS at the CFM (dashed curves) and CTM (solid curves) level. The same shift energy was applied for CFM and CTM calculations. (From top to bottom: (a) [Fe^II^(btz)_2_(bpy)]^2+^, (b) [Fe^II^(btz)_3_]^2+^, (c) [Fe^III^(btz)_3_]^3+^, (d)[Fe^III^(phtmeimb)_2_]^1+^, (e) [Fe^IV^(phtmeimb)_2_]^2+^).

The position of the e_g_ character peak in [Fe^II^(btz)_3_]^2+^ is higher by 0.66 eV compared to [Fe^II^(btz)_2_(bpy)]^2+^; see Table 3. This energy shift has multiple origins: one stems from the increase of the σ-donating capability of the ligands, which can destabilize the e_g_ energy level. This is in line with the argument that the btz ligand is a stronger σ donor than the bpy ligand. The replacement of all bpy ligands by btz ligands leads to *trans-*increased C–Fe–C angles of 179° compared to 159° in [Fe^II^(btz)_2_(bpy)]^2+^. The change of angle significantly improves the orthoaxiality of the [Fe^II^(btz)_3_]^2+^ compound. This improved orthoaxiality contributes to a larger ligand–field splitting and thus further destabilization of the e_g_ level. The AILFT calculations gave a 10Dq value of 2.7 and 3.2 eV for [Fe^II^(btz)_2_(bpy)]^2+^ and [Fe^II^(btz)_3_]^2+^, respectively, supporting this argumentation. The CFM calculations of both systems using the 10Dq from the AILFT calculations generate spectra that are very close to the measured spectra, but for a small shift of 0.3 eV of the main e_g_ peak, see Table 3. In general, one would expect a stronger σ electron donation capability of the three carbene ligands for [Fe^II^(btz)_3_]^2+^ compared to the two carbene ligands in [Fe^II^(btz)_2_(bpy)]^2+^. To reproduce the positions of the e_g_ character peak shift, we needed to include the LMCT configuration parameter for both [Fe^II^(btz)_2_(bpy)]^2+^ and [Fe^II^(btz)_3_]^2+^ but with a higher value for [Fe^II^(btz)_3_]^2+^. This is direct evidence for the stronger σ electron-donating capability of the three carbene ligands in [Fe^II^(btz)_3_]^2+^. A similar effect can be observed at the high energy side of the measured transitions into the L_3_ shell, where the difference between the calculated and measured is reduced after the inclusion of MLCT configurations. This suggests the π* accepting capability of these NHC ligands, see Figure 6. The comparison of the calculated and measured peak positions is summarized in Table 3.

In Table 2, we assign each active orbital a covalency percentage representing the amount of metal 3d character. At 100%, the orbital would behave as a pure metal orbital; at 50%, it would be equally shared with the ligand. Based on the orbital composition analysis, the π orbitals of [Fe^II^(btz)_3_]^2+^ have an average Fe 3d character of 78.1% in comparison to 83.6% for [Fe^II^(btz)_2_(bpy)]^2+^. This illustrates the strong π-back-bonding of the third btz ligand, which results in the more stable [Fe^III^(btz)_3_] configuration. The biggest difference is visible for the σ-e_g_ type orbitals, which can be understood as coming from the strength of the coupling. [Fe^II^(btz)_2_(bpy)]^2+^ has around 62.4%, while [Fe^II^(btz)_3_]^2+^ has 52.4% 3d character of the e_g_ type orbitals. This difference reflects the significantly improved geometric orthoaxiality from [Fe^II^(btz)_2_(bpy)]^2+^ to [Fe^II^(btz)_3_]^2+^, which increases the orbital overlap. In a simplified picture, the increased metal–ligand mixing could be interpreted as an increased delocalization of the metal electron density into the ligands and a subsequent decrease of electron density at the metal center and, hence, a reduced ability to screen the Fe 2p core hole in the core-excited state. This would result in a shift to higher incident energy of the corresponding signal in the metal L-edge XA spectrum, which is indeed observed.

**Table 2:** Orbital Covalency (in %) for the Iron Carbene Complexes Presented in This Paper, which Should be Understood as to what Percentage an Orbital has Metal 3d Character, where at 50%, it would be an Even Distribution between Metal and Ligand Character^a^

	π(3d_*xz*_)	π(3d_*yz*_)	π(3d_*xy*_)	σ(3d_*x*^2^–*y*^2^_)	σ(3d_*z*^2^_)
[Fe^II^(btz)_2_(bpy)]^2+^	86.3	82.6	82.0	62.4	62.3
[Fe^II^(btz)_3_]^2+^	76.5	81.2	76.5	54.2	54.2
[Fe^III^(btz)_3_]^3+^	86.5	94.3	86.5	56.3	56.2
[Fe^III^(phtmeimb)_2_]^1+^	67.0	94.3	85.6	56.8	56.9
[Fe^IV^(phtmeimb)_2_]^2+^	95.5	95.9	94.0	50.1	51.8

a Please see the SI section “representation of selected Fe 3d character active orbitals” for an illustration of the here-discussed orbitals.

Regarding the Fe^III^ complexes, both [Fe^III^(btz)_3_]^3+^ and [Fe^III^(phtmeimb)_2_]^1+^ have doublet ground states (t_2g_^5^e_g_^0^). The intensity of the first peak originates from 2p → t_2g_ transitions, which gives a 2p^5^t_2g_^6^e_g_^0^ final state in the valence shell. The mechanism that creates the observed absorption intensity can be confirmed from both a core-excited spin-state contribution and an orbital contribution analysis. The final spin-state contribution confirms that the states that contribute to the t_2g_ character peak are doublet core-excited states (Δ*S* = 0). The orbital contribution analysis clearly shows that the intensity comes from core excitations to the t_2g_ orbital (see Figure 5 panels c and d). While the overall shapes of the L-edge XAS of [Fe^III^(btz)_3_]^3+^ and [Fe^III^(phtmeimb)_2_]^1+^ are very similar, there are clear differences in the peak positions. The t_2g_ peak of [Fe^III^(phtmeimb)_2_]^1+^ is shifted by 0.20 eV compared to [Fe^III^(btz)_3_]^3+^, which is consistent with our previous iron K pre-edge XAS measurement.^20^ The wider energy spread of the multiplet structures of transitions into the valence orbitals with e_g_ character of Fe^III^ complexes compared to Fe^II^ complexes results in a significantly broadened e_g_ peak at ∼712 eV. This feature at ∼712 eV comes from the interactions between the excited electron in the e_g_ states with the electron in the singly occupied t_2g_ orbital. The RAS calculations allow for multiple excitations within the active space and can describe the electron–electron interactions in the valence shell and nicely reproduce the spectral feature.

The energy of the feature that corresponds to transitions into the orbitals with the e_g_ character being shifted to higher energies by 0.25 eV for [Fe^III^(phtmeimb)_2_]^1+^ compared to [Fe^III^(btz)_3_]^3+^ if determined by the energy of the maximum height or by 0.14 eV if determined through the first moment of the intensity distribution. This is expected as the [phtmeimb]^1–^ ligand has a more pronounced σ-donating capability, which can destabilize/shift the energy of the e_g_ orbitals. The AILFT calculations gave a 10Dq value of 3.4 eV for [Fe^III^(btz)_3_]^3+^ and a slightly larger 10Dq value of 3.8 eV for [Fe^III^(phtmeimb)_2_]^1+^. In the CFM calculations, we found that the first e_g_ peak is shifted to slightly higher energy when comparing [Fe^III^(btz)_3_]^3+^ to [Fe^III^(phtmeimb)_2_]^1+^. The CFM calculations, in general, show a smaller splitting (see Table 3) between the absorption peaks corresponding to the t_2g_ and e_g_ orbitals for both Fe^III^ complexes due to the lack of metal–ligand orbital mixing. The estimated positions of the calculated features come closer to the measured position if the parameter describing the metal–ligand orbital mixing is increased, particularly when including the σ donating LMCT configuration. Combining the slightly lower energy of the t_2g_ peak with the slightly higher energy of the e_g_ peak in [Fe^III^(phtmeimb)_2_]^1+^ shows a larger ligand–field splitting in this complex, which is consistent with the AILFT calculated values. The destabilized e_g_ character orbital shows higher energy for the metal-centered (MC) valence-excited states, such as ^4^MC and ^6^MC states in [Fe^III^(phtmeimb)_2_]^1+^. Again, the lower t_2g_ peak indicates that [Fe^III^(phtmeimb)_2_]^1+^ has a π orbital at lower energies with metal t_2g_ character, which can be reflected in a lower-energy ligand-to-metal charge-transfer (LMCT) state. Our previous work has shown that the ^4^MC and ^6^MC states of [Fe^III^(phtmeimb)_2_]^1+^ are destabilized compared to those of [Fe^III^(btz)_3_]^3+^.^15^ The ^2^LMCT energy of [Fe^III^(phtmeimb)_2_]^1+^ is 2.13 eV, about 0.17 eV lower than for [Fe^III^(btz)_3_]^3+^, which is 2.30 eV. The increased energies of the MC state, together with the decreased energy of the ^2^LMCT state, gives an increased activation barrier for the decay of the ^2^LMCT state into the MC state and consequently contributes to the increase of the experimentally observed lifetime of the charge-separated ^2^LMCT state.^13,15^ The t_2g_ and the e_g_ character peaks in the iron L-edge XA spectrum well reproduce this larger splitting between the t_2g_ and the e_g_ metal character molecular orbitals in [Fe^III^(phtmeimb)_2_]^1+^.

**Table 3:** Experimentally and Computationally Determined (Maximum Point) Photon Energies for the Resonances at the Iron L_3_ Edge^a^

	experimental		RAS		CFM		CTM	
	t_2g_	e_g_	t_2g_	e_g_	t_2g_	e_g_	t_2g_	e_g_
[Fe^II^(btz)_2_(bpy)]^2+^		710.64		710.54		710.56		710.70
[Fe^II^(btz)_3_]^2+^		711.30		711.67		710.82		711.25
[Fe^III^(btz)_3_]^3+^	707.52	712.01	707.67	712.20	707.63	711.28	707.50	712.05
[Fe^III^(phtmeimb)_2_]^1+^	707.32	712.26	707.37	712.12	707.46	711.61	707.21	712.29
[Fe^IV^(phtmeimb)_2_]^2+^	707.95	712.10	708.29	713.28	707.52	711.02	707.72	712.28

a All values are given in eV.

The complex [Fe^IV^(phtmeimb)_2_]^2+^ has an intermediate-spin ground state (*S* = 1) with a valence electronic configuration of t_2g_^4^e_g_^0^. There are two singly occupied t_2g_ orbitals and one fully occupied t_2g_ orbital, while the two e_g_ character orbitals are empty. In the L-edge XA spectrum, a well-separated t_2g_ peak is visible, which is significantly stronger than the corresponding peak in the spectra of the Fe^III^ complexes. One of the main contributions to this difference is the addition of a t_2g_ hole. [Fe^IV^(phtmeimb)_2_]^2+^ also has a broad e_g_ character peak due to the interaction between the excited electron in the e_g_ orbitals and the valence electrons in the t_2g_ orbitals. The oxidation change from [Fe^III^(phtmeimb)_2_]^1+^ to [Fe^IV^(phtmeimb)_2_]^2+^ results in higher energy of the t_2g_ orbital, while the shift for the e_g_ orbitals is hidden behind the broad multiplet structure and broadened features, see Table 3. As there is also a spin state change when the oxidation state changes, it is complicated to directly analyze the edge shift between [Fe^III^(phtmeimb)_2_]^1+^ and [Fe^IV^(phtmeimb)_2_]^2+^. Table 3 summarizes the calculated and experimental positions of the main t_2g_ and e_g_ features. The converged AILFT calculation for the [Fe^IV^(phtmeimb)_2_]^2+^ complex predicted the wrong spin state as the ground state, which might stem from the restriction of only five metal character orbitals that could be included in the calculation. For an open-shell ground state with multiple singly occupied and also empty orbitals, more correlated ligand character orbitals and sometimes the double shell correlating orbitals are usually required.

To still use reasonable CTM parameters for the calculation of the [Fe^IV^(phtmeimb)_2_]^2+^ spectra, we adapted the obtained parameters from the reduced complex [Fe^III^(phtmeimb)_2_]^1+^ and obtained significantly worse agreement between the experiment and the calculations because of this. Unsurprisingly, fitting parameters needed for a complicated open-shell system without evident symmetry is difficult. *Ab initio* methods such as the RAS have a clear advantage in these systems, as they suffer less from these limitations.

### N K-Edge XA Spectra: Probing the Ligand

**Figure 7:**
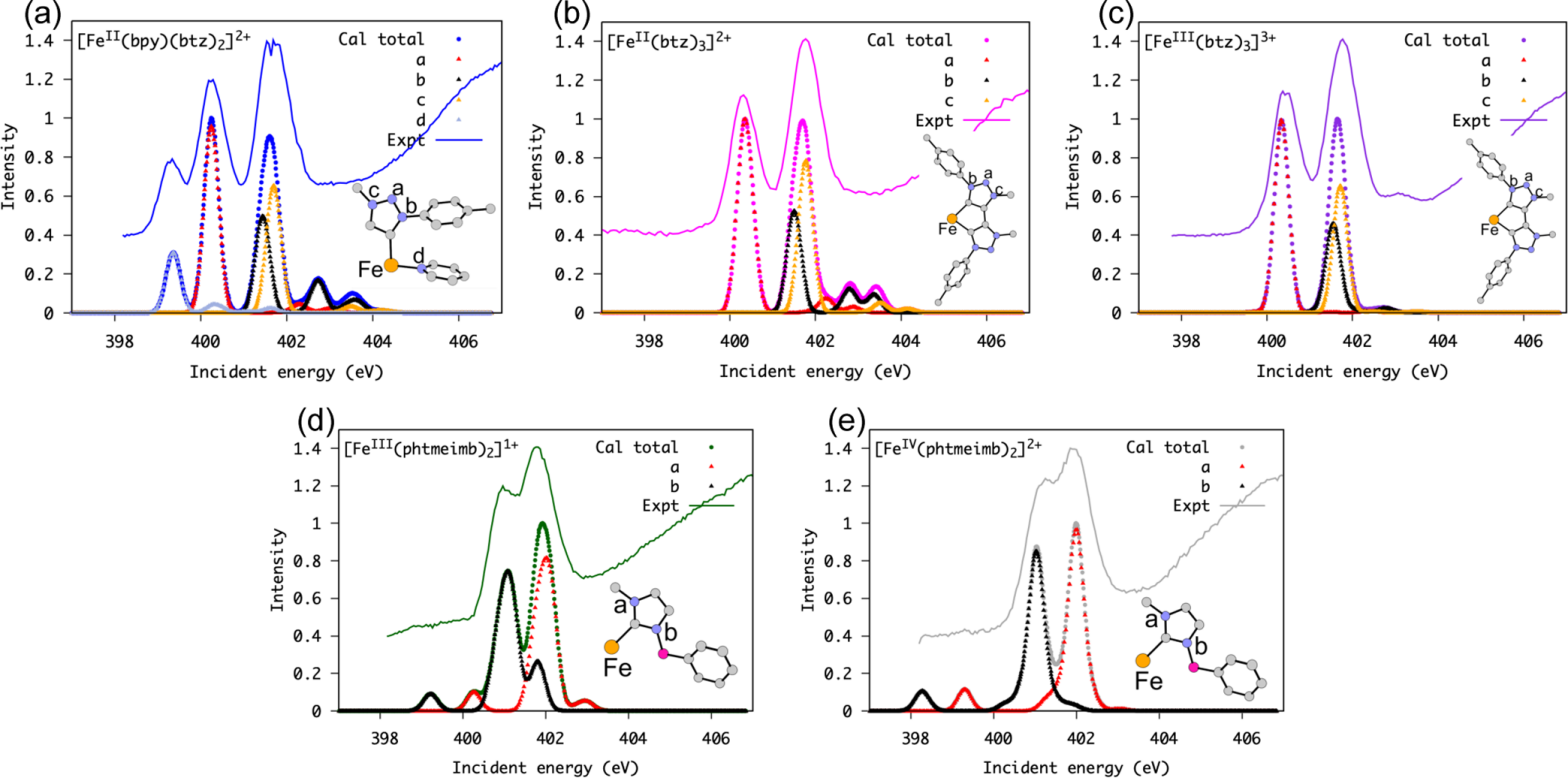
Experimental and calculated XA spectra for all complexes calculated with TD-DFT. (a) ([Fe^II^(btz)_2_(bpy)]^2+^, (b) [Fe^II^(btz)_3_]^2+^, (c) [Fe^III^(btz)_3_]^3+^, (d)[Fe^III^(phtmeimb)_2_]^1+^, (e) [Fe^IV^(phtmeimb)_2_]^2+^).

One obtains a complementary view of the investigated complexes by probing the nitrogen atoms and thus the ligands. In Figure 7, we show the calculated and experimental N K-edge XA spectra for each of the complexes. In this figure, the calculated spectra were decomposed into the contributions from each N atom.

In the spectrum of [Fe^II^(btz)_2_(bpy)]^2+^ in Figure 7 a, the first peak at ∼399.3 eV is attributed to an excitation from the N 1s orbital into the π* molecular orbital, which is almost entirely located on the bpy ligand. That π* molecular orbital corresponds to the LUMO of [Fe^II^(btz)_2_(bpy)]^2+^; see the selected molecular orbitals in Figure SI 18. The second experimentally resolvable feature originates from excitation into the corresponding π* molecular orbitals on the btz ligands. The double peak structure with peaks at ∼400.2 and ∼401.8 eV arises from the difference in the N 1s core-level energy of the N_a_ vs the N_b_/N_c_ atoms (see inset molecule schemes in Figure 7). The N 1s energy difference between the N_a_ vs the N_b_/N_c_ atoms has previously been confirmed by X-ray photoelectron spectroscopy measurements.^58^ The first peak in [Fe^II^(btz)_2_(bpy)]^2+^ is not present in the N K-edge XA spectra of [Fe^II^(btz)_3_]^2+^, which again confirms the bpy character of the first peak in [Fe^II^(btz)_2_(bpy)]^2+^. The N K-edge XA spectra of [Fe^II^(btz)_3_]^2+^ and [Fe^III^(btz)_3_]^3+^ are very similar to each other. Both [Fe^II^(btz)_3_]^2+^ and [Fe^III^(btz)_3_]^3+^ have two separated peaks due to excitation on the N_a_ and N_b_/N_c_ atoms, respectively, similar to what is seen for the [Fe^II^(btz)_2_(bpy)]^2+^. One could speculate that this clear difference between the a and b/c features in the spectrum of [Fe^II^(btz)_3_]^2+^ could allow one to probe the influence of nodal structure predicted by DFT with time-resolved tools.^58^ This separation of the nitrogen transitions is apparent in the spectra of both the [Fe^II^(btz)_3_]^2+^ and [Fe^III^(btz)_3_]^3+^ complexes, with very little change, apart from slight relative shifts in the N_b_ and N_c_ peaks when going from Fe^II^ to Fe^III^. This is consistent with our previous assignments.^58,59^

In the spectra of the [Fe^III^(phtmeimb)_2_]^1+^ and [Fe^IV^(phtmeimb)_2_]^2+^ complexes, the two main peaks are predominantly due to excitations from the two different N atoms in the ligands into the same π*-derived molecular orbital. The spectra of [Fe^III^(phtmeimb)_2_]^1+^ and [Fe^IV^(phtmeimb)_2_]^2+^ exhibit separated and distinct pre-edge features. The calculations indicate that the features originate from N 1s excitations to singly occupied molecular orbitals (SOMOs), which are mainly metal t_2g_ characters with some intermixing of N characters, cf. the orbital file in the SI. The intensities of the pre-edge originate from the N 1s electron excitation from the different N atoms (N_a_ and N_b_) into the SOMO. Interestingly, a similar pre-edge feature is not observable for [Fe^III^(btz)_3_]^3+^.

The orbital analysis shows that the SOMOs with metal t_2g_ character for the complexes [Fe^III^(phtmeimb)_2_]^1+^ and [Fe^IV^(phtmeimb)_2_]^2+^ have an energy that is respectively 2.3 and 3.1 eV lower than the ligand-character π* orbitals on the imidazole ligand. The metal character of the SOMO orbitals in [Fe^III^(btz)_3_]^3+^ results in a much smaller shift of only 0.3 eV compared to that of the π* orbital. In [Fe^III^(btz)_3_]^3+^, the N 1*s* electron excitations into the SOMO’s are coupled to excitations to the other π* orbitals, and the otherwise visible pre-edge feature merges with the intense main peak, see Figure 7.

Besides the difference in the pre-edge feature in the N K-edge XA spectrum, the main peaks in [Fe^III/IV^(phtmeimb)_2_]^1+/2+^ are located at higher energies than those in [Fe^II/III^(btz)_3_]^2+/3+^. This is in agreement with the general consideration that the btz ligand has a much stronger π-accepting capability than the [phtmeimb]^1–^ ligand. We have drawn the same conclusion when discussing the metal L-edge XAS. This difference in the π-accepting capability was also reflected in the CTM calculations of the L-edge XAS, where a MLCT configuration with a relatively high energy and a smaller overlap was used for [Fe^III/IV^(phtmeimb)_2_]^1+/2+^. This suggests that the main peak shift in the N 1*s* XA spectrum can be used as a probe of the π-accepting capability difference between [Fe^II/III^(btz)_3_]^2+/3+^ and [Fe^III/IV^(phtmeimb)_2_]^1+/2+^. In other words, the shift and strength of the features in the range 399 to 400 eV is indicative of the strength of the π-back-bonding and could be used in future studies to characterize it.

## Summary

The XA spectra at the Fe L-edge and N K-edge have been used to selectively probe both the metal centers and ligands of a series of iron carbene complexes of varying oxidation states and ligand–field interactions. The experimental spectral features are interpreted through RAS and multiplet calculations of iron L-edge XAS, together with TDDFT calculations of N K-edge XAS. The Fe L-edge XA spectra provide a direct measurement of the ligand field-induced splitting of the Fe 3d into t_2g_ and e_g_ orbitals. We observed the destabilization of the e_g_ orbitals with increasing σ-donating strength of the ligand. Through the calculations of ligand field parameters and multiplet calculations of L-edge XAS, we individually evaluated the important spectral effects, such as 10Dq, LMCT, and MLCT.

In the d^6^ systems [Fe^II^(btz)_2_(bpy)]^2+^ and [Fe^II^(btz)_3_]^2+^, the complex [Fe^II^(btz)_3_]^2+^ has the larger 10Dq value according to the AILFM calculations, while the position of the e_g_ character peak in L-edge XAS is also affected by the ligand character. By tuning (and thus measuring) the metal–ligand mixing configurations (LMCT and MLCT) in the CTM calculations, the experimental features can be well reproduced in terms of the e_g_ position and the features at the higher energy in the L_3_ edge. A similar method led in the two d^5^ systems [Fe^III^(btz)_3_]^3+^ and [Fe^III^(phtmeimb)_2_]^1+^, to a better estimation of the splitting between t_2g_ and e_g_. We have used *ab initio* RAS calculations to estimate the peak intensity and positions, which was complemented by the AILFT and multiplet calculations. For most of the complexes, we successfully reproduced the measured spectra and extracted the ligand field parameters from the fits.

In N K-edge XAS, we show resonant transitions from the different ligand moieties into the π* structures. The energies of these peaks are directly related to the π-accepting ability of the different ligands. We have discussed the transition from ligand N 1*s* orbitals into metal t_2g_ character orbitals as a direct probe of π-back-bonding. Experimentally, these features will allow future exploration in time-resolved experiments.

Overall, with an X-ray probe of the metal and ligand, we demonstrate that the ligand σ donating capability follows a clear trend [phtmeimb]^1–^ > btz > bpy. The π accepting capability has little difference in [Fe^II^(btz)_2_(bpy)]^2+^ and [Fe^II^(btz)_3_]^2+^. This is reflected in only a minimal shift in the N K-edge features. Based upon the CTM calculations, we find that the btz ligand is a stronger π accepting ligand than [phtmeimb]^1–^. Overall, we find that a combined approach using different levels of theory yielded a significantly more robust description of the measured spectra. Combining XA spectra from both the ligand and the metal center, allowed information to be extracted without access to complementary and often (due to access) more challenging experimental techniques.

## Supporting Information Available

Additional experimental details: expanded spectral comparison; expanded dependency of calculation parameters; dependency on the basis set; dependency on the number of core-excited states PDOS of the ground state and core-ionized state in Z + 1 approximation; representation of selected Fe 3d character active orbitals; and representation of selected DFT type orbitals (PDF)
