# Supplementary material for: Using Iron L-Edge and Nitrogen K-Edge X-ray Absorption Spectroscopy to Improve the Understanding of the Electronic Structure of Iron Carbene Complexes
Source: Inorg Chem. 2024 Jun 27;63(27):12457–68. doi: 10.1021/acs.inorgchem.4c01026 (PMC11234367; doi:10.1021/acs.inorgchem.4c01026)
Supplement: Supplementary file 1 — ic4c01026_si_001.pdf [file ic4c01026_si_001.pdf]

Supporting Information to: Using iron L-edge and nitrogen K-edge x-ray absorption spectroscopy to improve the understanding of the electronic structure of iron carbene complexes

Meiyuan Guo,[†] Robert Temperton,[‡] Giulio D'Acunto,[¶,§,||] Niclas Johansson,[‡] Rosemary Jones,[¶,§] Karsten Handrup,[‡] Sven Ringelband,[†] Om Prakash,[⊥] Hao Fan,[⊥] Lisa H. M. de Groot,[⊥] Valtýr Freyr Hlynsson,[⊥] Simon Kaufhold,[⊥] Olga Gordivska,[⊥] Nicolás Velásquez González,[†] Kenneth Wärnmark,[⊥,§] Joachim Schnadt,[¶,‡,§] Petter Persson,[#,§] and Jens Uhlig[*,†,@,§]

†*Division of Chemical Physics, Department of Chemistry, Lund University, 22100, Lund, Sweden*

‡*MAX IV Laboratory, Lund University, 22100, Lund, Sweden*

¶*Division of Synchrotron Radiation Research, Department of Physics, Lund University, 22100, Lund, Sweden*

§*NanoLund, Lund University, 22100, Lund, Sweden*

||*Department of Chemical Engineering, Stanford University, Stanford, CA 94305, USA*

⊥*Centre for Analysis and Synthesis (CAS), Department of Chemistry, Lund University, 22100, Lund, Sweden*

#*Division of Computational Chemistry, Department of Chemistry, Lund University, Sweden*

@*LINXS Institute of Advanced Neutron and X-Ray Science, Lund University, 22370, Lund, Sweden*

E-mail: jens.uhlig@chemphys.lu.se

Contents:

1. Expanded spectral comparison

2. Expanded dependency of calculation parameters

3. Dependency on the basis set

4. Dependency on the number of core-excited states

5. PDOS of the ground state and core-ionized state in Z+1 approximation

6. Representation of selected Fe 3d character active orbitals

7. Representation of selected DFT type orbitals

Expanded spectral comparison

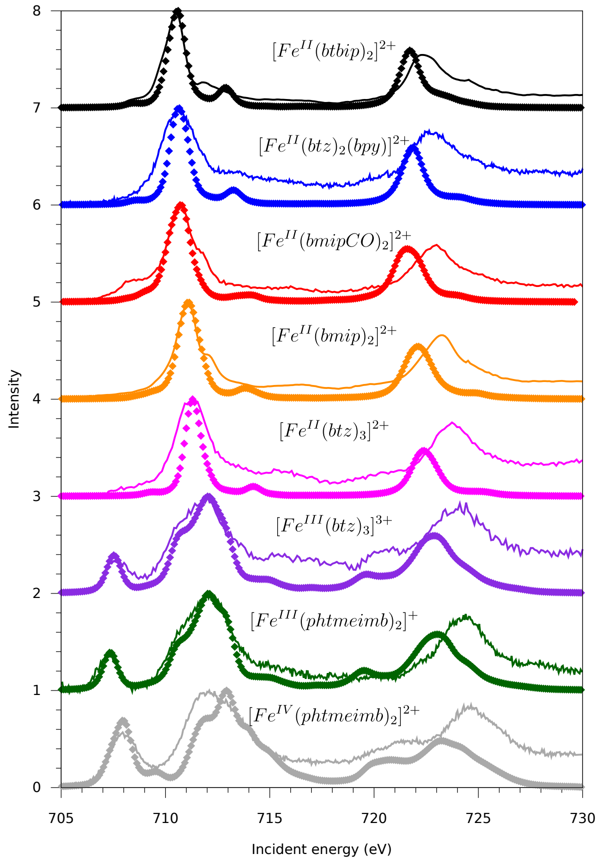

Figure SI 1: Expanded summary including the previously published molecules. bmip = 2,6-bis(3-methyl-imidazole-1-ylidine)pyridine, bmipCO = (1,1'-(4-carboxypyridine-2,6-diyl)bis(3-methylimidazol-2-ylidene)), btbip = 2,6-bis(3-tert-butyl-imidazol-1-ylidene)pyridine.

Expanded dependency of calculation parameters

Dependency on the basis set

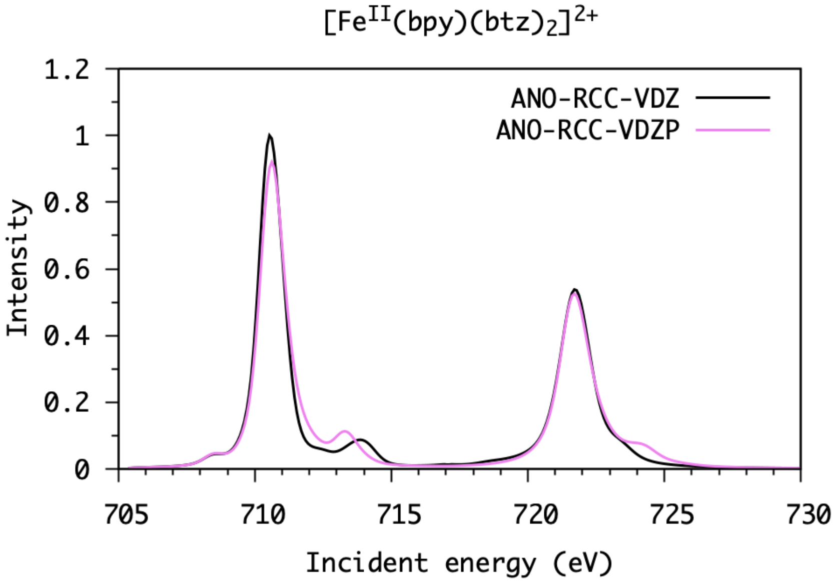

Figure SI 2: The Fe L-edge XAS spectral feature dependence on basis set.

Dependency on the number of core-excited states

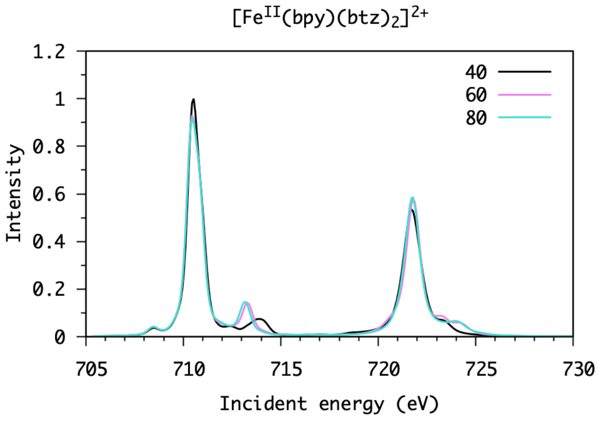

Figure SI 3: The Fe L-edge XAS spectral feature dependence on the number of core excited state.

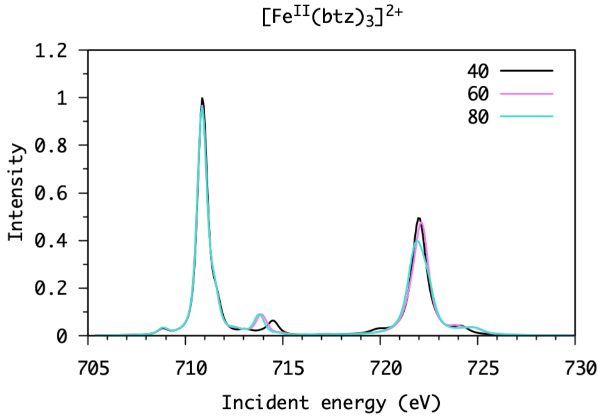

Figure SI 4: The Fe L-edge XAS spectral feature dependence on the number of core excited state.

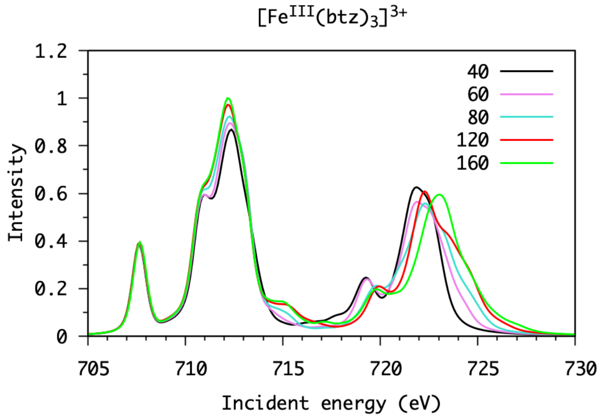

Figure SI 5: The Fe L-edge XAS spectral feature dependence on the number of core excited state.

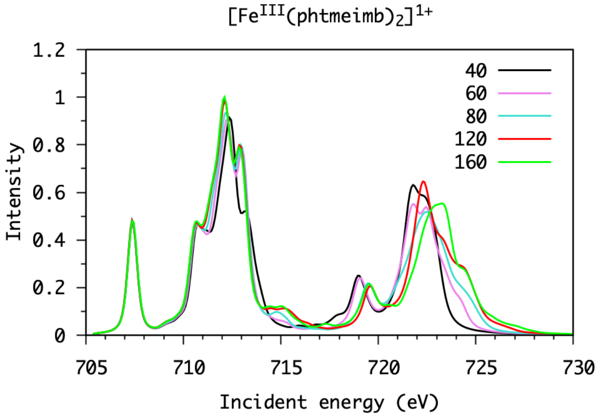

Figure SI 6: The Fe L-edge XAS spectral feature dependence on the number of core excited state.

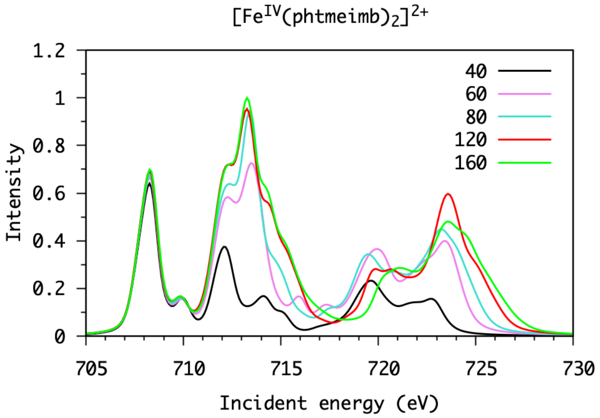

Figure SI 7: The Fe L-edge XAS spectral feature dependence on the number of core excited state.

PDOS of the ground state and core-ionized state in Z+1 approximation

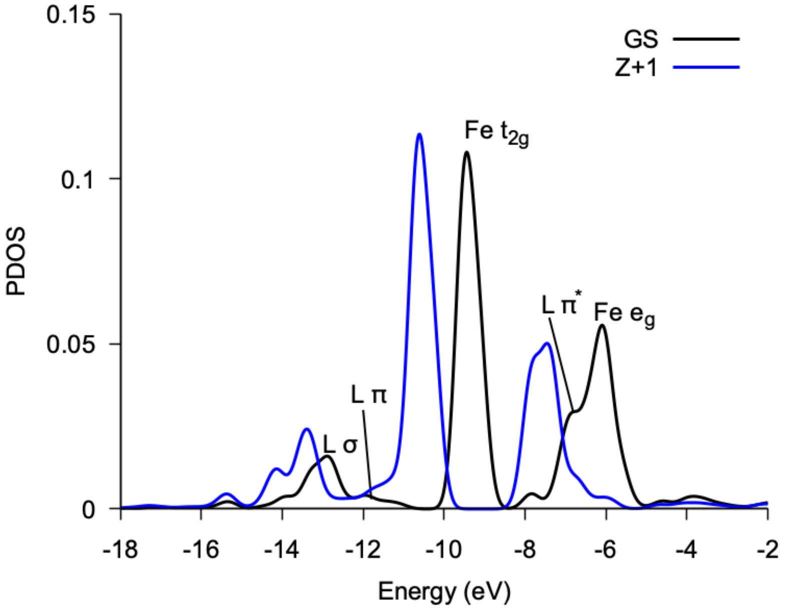

Figure SI 8: Calculated PDOS of the ground state and core-ionized state in Z+1 approximation of $[Fe^{II}(btz)_2(bpy)]^{2+}$. 0.5 eV FWHM Gaussian broadening is used.

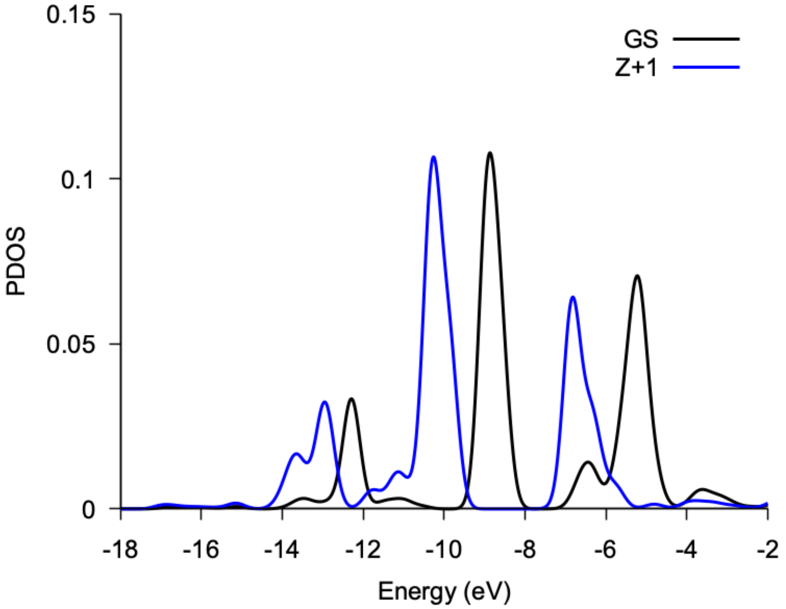

Figure SI 9: Calculated PDOS of the ground state and core-ionized state in Z+1 approximation of $[Fe^{II}(btz)_3]^{2+}$. 0.5 eV FWHM Gaussian broadening is used.

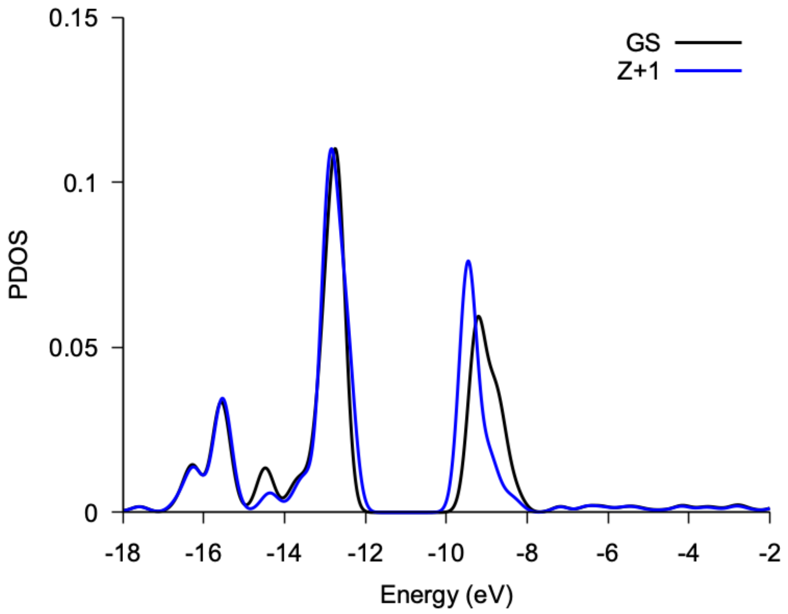

Figure SI 10: Calculated PDOS of the ground state and core-ionized state in Z+1 approximation of $[Fe^{III}(btz)_3]^{3+}$. 0.5 eV FWHM Gaussian broadening is used.

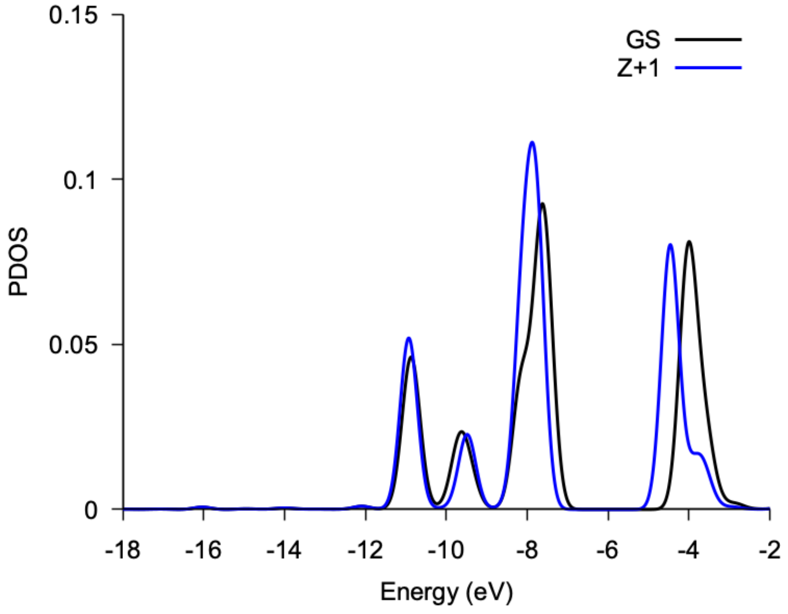

Figure SI 11: Calculated PDOS of the ground state and core-ionized state in Z+1 approximation of $[Fe^{III}(phtmeimb)_2]^{+}$. 0.5 eV FWHM Gaussian broadening is used.

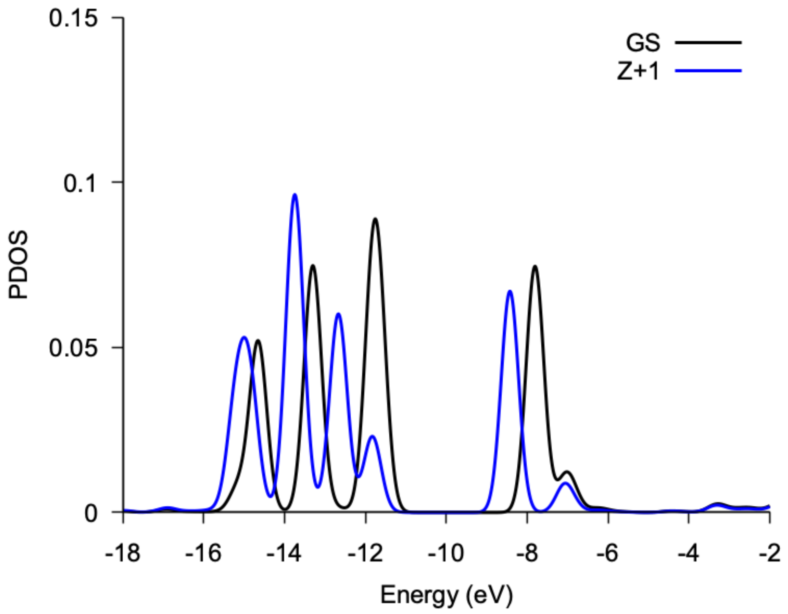

Figure SI 12: Calculated PDOS of the ground state and core-ionized state in Z+1 approximation of $[Fe^{IV}(phtmeimb)_2]^{2+}$. 0.5 eV FWHM Gaussian broadening is used.

Representation of selected Fe 3d character active orbitals

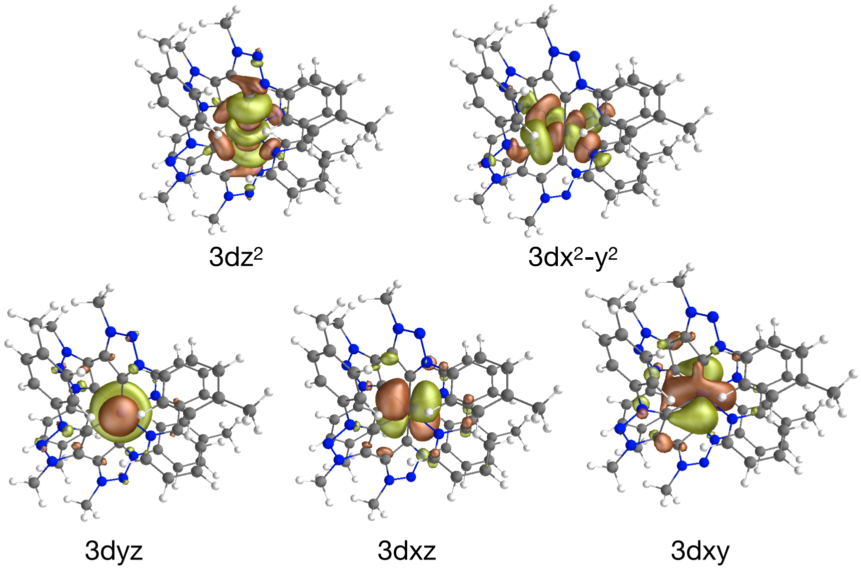

Figure SI 13: Selected molecular orbitals of $[Fe^{II}(btz)_2(bpy)]^{2+}$used in the iron L-edge XAS calculation.

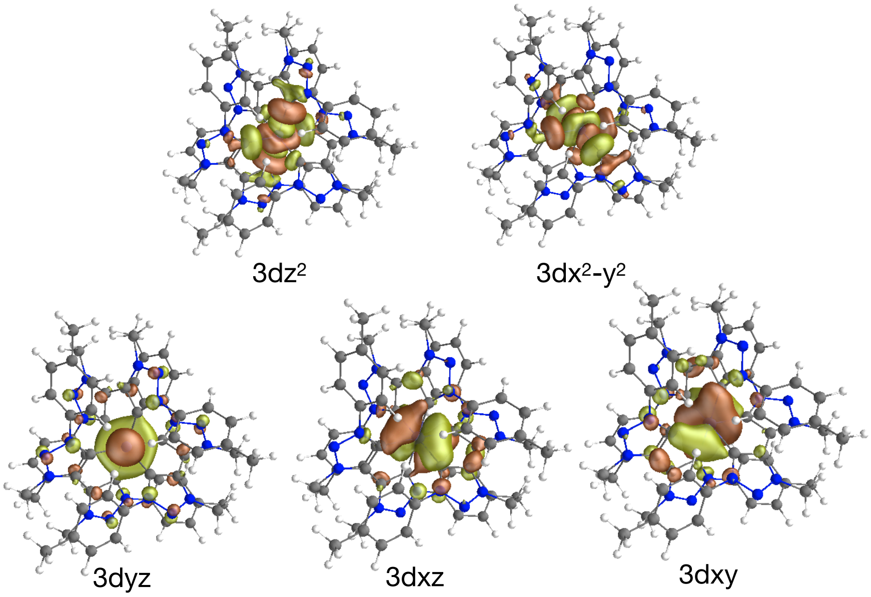

Figure SI 14: Selected molecular orbitals of $[Fe^{II}(btz)_3]^{2+}$used in the iron L-edge XAS calculation.

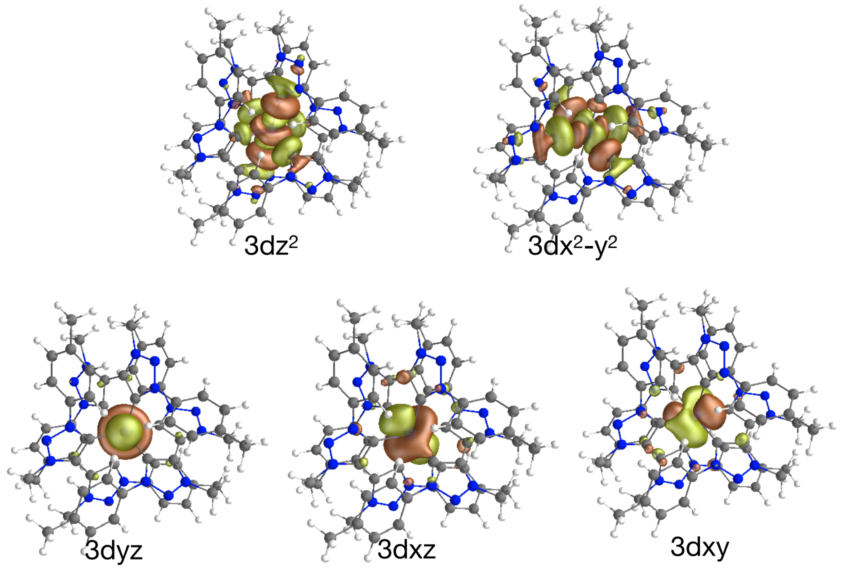

Figure SI 15: Selected molecular orbitals of $[Fe^{III}(btz)_3]^{3+}$used in the iron L-edge XAS calculation.

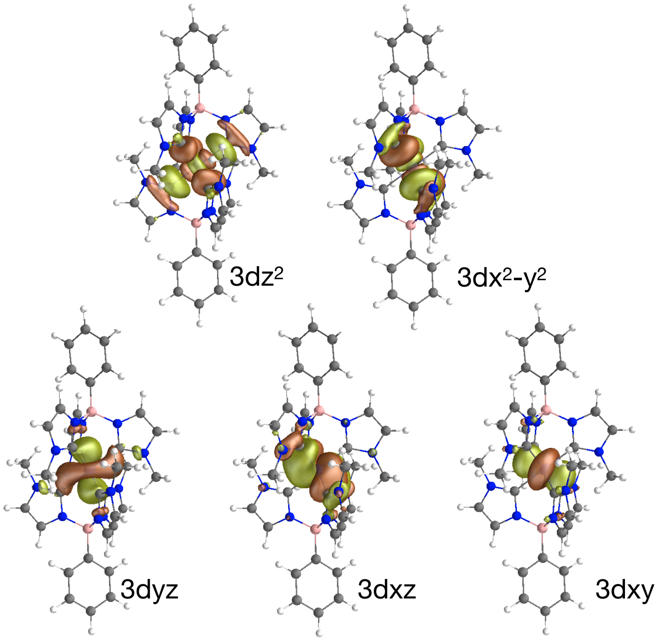

Figure SI 16: Selected molecular orbitals of $[Fe^{III}(phtmeimb)_2]^+$used in the iron L-edge XAS calculation.

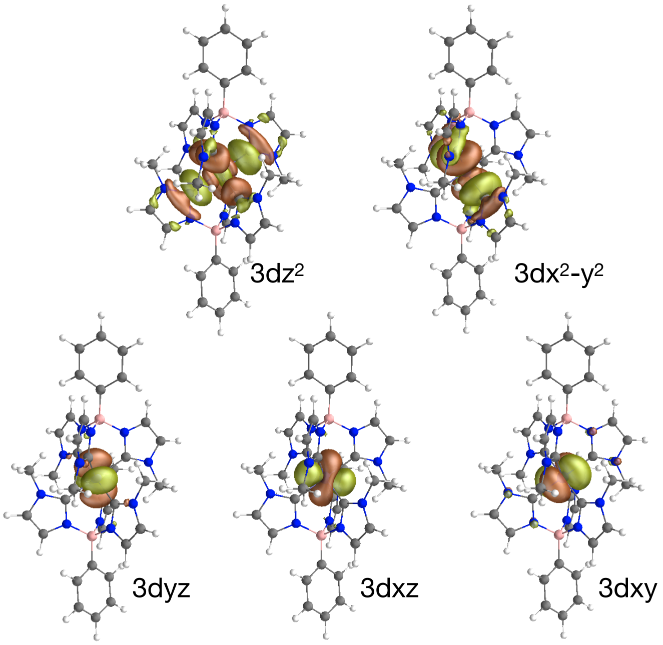

Figure SI 17: Selected molecular orbitals of $[Fe^{IV}(phtmeimb)_2]^{2+}$used in the iron L-edge XAS calculation.

Representation of selected DFT type orbitals

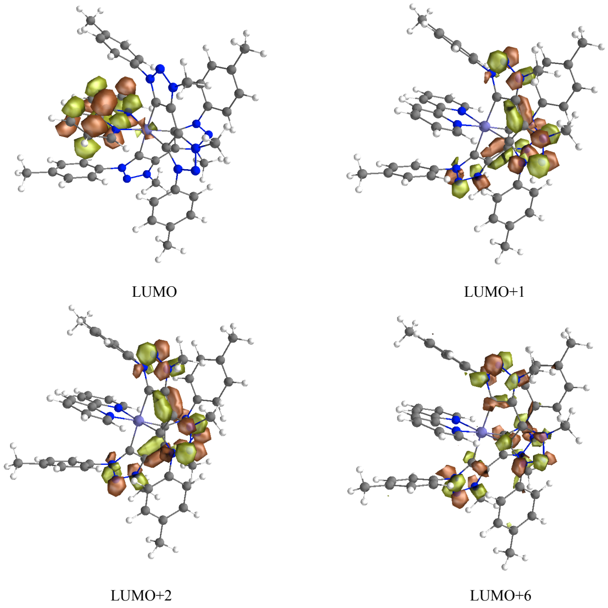

Figure SI 18: Selected molecular orbitals of $[Fe^{II}(btz)_2(bpy)]^{2+}$relevant for the discussion of nitrogen K-edge XAS.

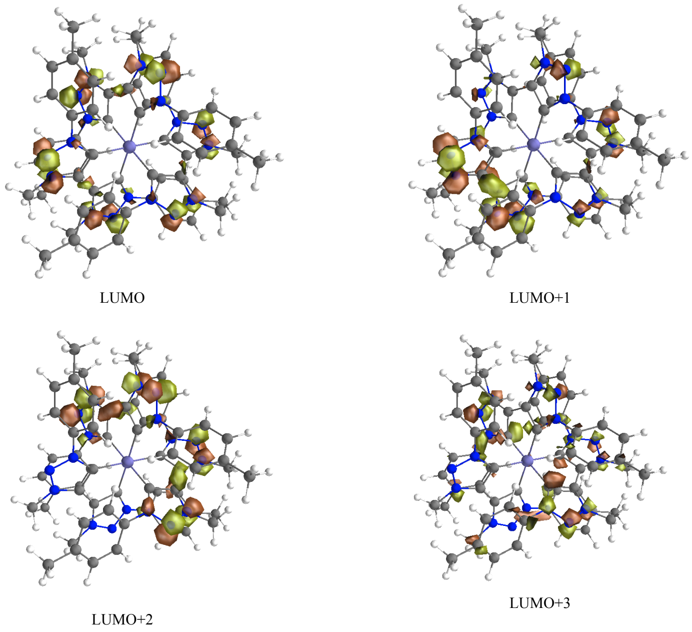

Figure SI 19: Selected molecular orbitals of $[Fe^{II}(btz)_3]^{2+}$relevant for the discussion of nitrogen K-edge XAS.

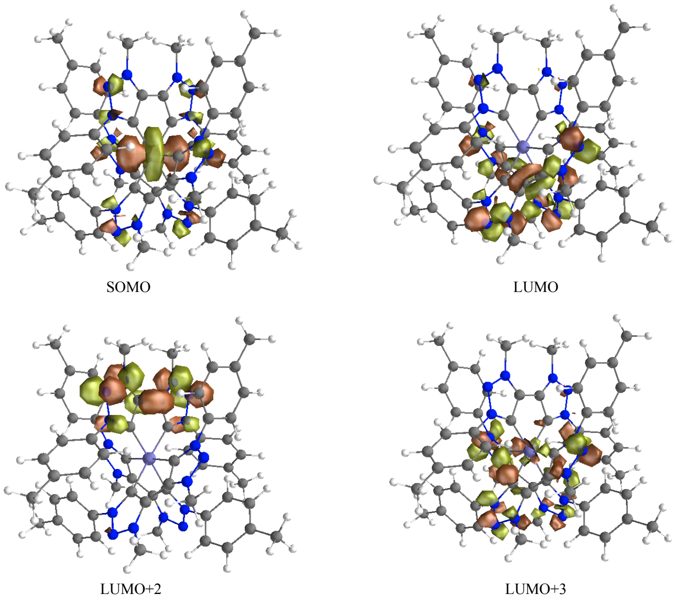

Figure SI 20: Selected molecular orbitals of $[Fe^{III}(btz)_3]^{3+}$relevant for the discussion of nitrogen K-edge XAS.

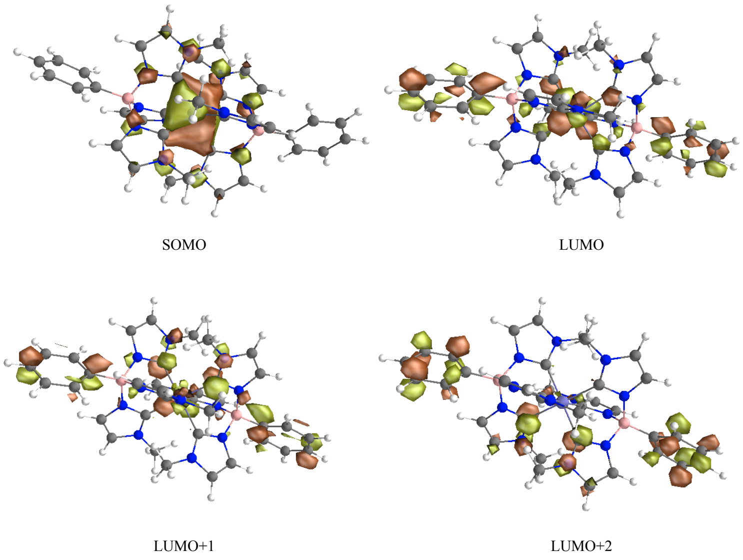

Figure SI 21: Selected molecular orbitals of $[Fe^{III}(phtmeimb)_2]^{+}$relevant for the discussion of nitrogen K-edge XAS.

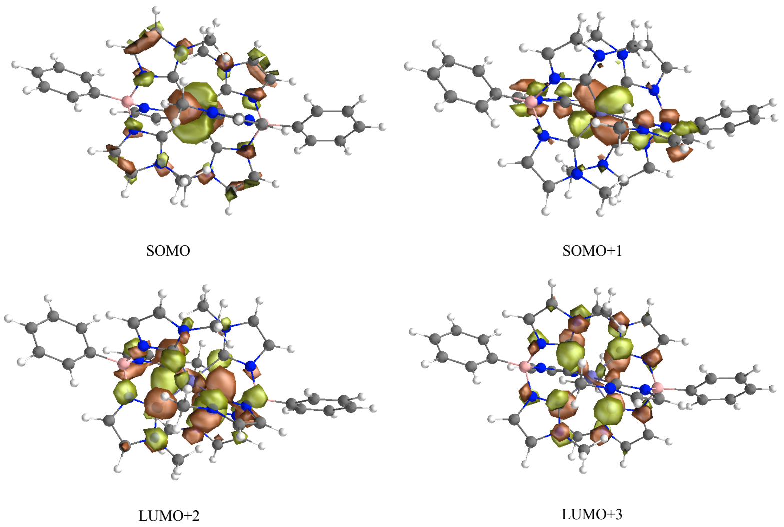

Figure SI 22: Selected molecular orbitals of $[Fe^{IV}(phtmeimb)_2]^{2+}$relevant for the discussion of nitrogen K-edge XAS.
